# Most colitis associated carcinomas lack expression of LGR5: a preliminary study with implications for unique pathways of carcinogenesis compared to sporadic colorectal carcinoma

**DOI:** 10.1186/s12885-021-07835-3

**Published:** 2021-02-04

**Authors:** Mai Iwaya, Hiroyoshi Ota, Tomoyuki Nakajima, Takeshi Uehara, Robert Riddell, James Conner

**Affiliations:** 1 Department of Pathology and Laboratory Medicine, Mount Sinai Hospital, Toronto, Canada; 2 Department of Laboratory Medicine and Pathobiology, University of Toronto, Toronto, Canada; 3 Department of Laboratory Medicine, Shinshu University Hospital, 3-1-1 Asahi, Matsumoto, Nagano Japan; 4 Department of Clinical Laboratory Sciences, School of Health Sciences, Shinshu University, Matsumoto, Japan

**Keywords:** Inflammatory bowel disease, Ulcerative colitis, Crohn’s disease, Colitis associated colorectal carcinoma, LGR5

## Abstract

**Background:**

Leucine-rich repeat-containing G-protein-coupled receptor 5 (LGR5), a component of the Wnt receptor complex, is thought to lineage label gastric and intestinal stem cells. *LGR5* expression is increased in colorectal carcinoma (CRC) compared to normal tissue. Colitis associated colorectal adenocarcinoma (CAC) often shows distinct morphologic and molecular phenotypes compared to sporadic cases. However, the expression profile of LGR5, and by extension the potential role of an intestinal stem cell phenotype, has not been well described in a series of human CAC.

**Method:**

RNA in situ hybridization (ISH) for *LGR5* expression on 30 CACs (12 cases with conventional morphology and 18 cases with non-conventional type morphology) from 29 inflammatory bowel disease (IBD) patients was performed and compared the expression profile to a control group of 10 sporadic CRCs. Immunohistochemistry for beta-catenin and SATB2 was performed on the 30 CACs.

**Result:**

*LGR5* was positive in 30% (9/30) of CAC cases and 90% (9/10) of sporadic CRCs (*p* = 0.002). A large majority (89%) of *LGR5* positive CACs were of the conventional histologic type, and conventional type CAC showed a significantly higher *LGR5* score (median 3.0; interquartile range 1.75–3.25) than non-conventional type CAC (median 1.5; interquartile range 1.00–2.00) (*p* = 0.034). CAC with conventional morphology did have a lower level of LGR5 expression than sporadic CRC. Sporadic CRCs showed a significantly higher LGR5 level score than non-conventional type CACs (*p* < 0.001). Nuclear translocation of beta-catenin was strongly associated with *LGR5* expression (*p* = 0.003), however no significant association was identified between SATB2 expression and *LGR5* expression status in CACs.

**Conclusion:**

These findings suggest that the wider spectrum of tumor morphology in CAC may be associated with absence of a *LGR5*-expressing intestinal stem cell phenotype.

## Background

Inflammatory bowel disease (IBD) is a chronic relapsing–remitting disorder of the gastrointestinal tract, and for patients with long-standing colonic inflammation, colorectal adenocarcinoma (CRC) is a recognized complication, leading to routine periodic surveillance of patients with colitis for dysplasia and carcinoma. One meta-analysis estimated that the risk of colitis associated carcinoma (CAC) in patients with IBD is 2% by 10 years after initial diagnosis, and increases to 8% at 20 years and 18% at 30 years after colitis onset [1]. However more recent figures are lower than this, likely the results of better treatment reducing the risk [2, 3].

Leucine-rich repeat-containing G-protein-coupled receptor 5 (LGR5), a wingless-type mouse mammary tumor virus integration site family (Wnt) target gene that functions as a receptor for Wnt agonist R-spondins (RSPOs). It was identified as a selective marker of crypt base columnar cells, and these LGR5-positive crypt base columnar cells were demonstrated to be self-renewing, multipotent adult intestinal stem cells [4]. Increased *LGR5* expression in human sporadic colorectal adenomas and cancers [5–8] and in murine models [9, 10] have been well described, however, the *LGR5* expression profile has not been described in a series of CAC in human.

Recent studies have highlighted genetic, morphologic, and immunohistochemical differences between CAC and sporadic CRC, and suggested that chronic inflammation may lead to unique genomic changes that increase the risk for CAC [11, 12]. Carcinogenic mutations probably first start accumulating in the long-lived stem cell lineages of the crypt, as the lifespan of non-stem cells is too short for them to acquire the necessary mutations before being shed.

To our knowledge, a comparison of *LGR5* expression in CAC and sporadic CRC has not been performed. Here, a series of CAC cases was compared with sporadic CRC for morphologic features and the expression of *LGR5*.

## Methods

Study approval was obtained from the Research Ethics Board at Mount Sinai Hospital.

Thirty cases of surgically resected colitis associated primary colorectal adenocarcinoma from 29 patients between 2011 and 2016 were retrieved from the surgical pathology archives at Sinai Health System. Patients who underwent neoadjuvant therapy were excluded. 10 cases of surgically resected primary CRC from patients without inflammatory bowel disease or any known hereditary cancer syndrome, hence forth termed sporadic CRC were identified as a control group. All H&E sections were reviewed at a multiheaded microscope by four gastrointestinal pathologists (MI, HO, RR, and JC) and each invasive carcinoma was subclassified by consensus into one of five morphologic subtypes: conventional, mucinous, serrated, low grade tubuloglandular (LGTG) and others [13].

One representative paraffin block of tumor was selected in each case for RNA in situ hybridization (ISH). Detection of *LGR5* mRNA was performed using the RNAscope® kit (Advanced Cell Diagnostics, Hayward, CA, USA) according to the manufacturer’s instructions using unstained sample tissue slides. Briefly, tissue sections were pretreated by heating and protease application prior to hybridization with an *LGR5*-specific probe. A detailed procedure has been described in an earlier publication [14].

**Fig. 1:**
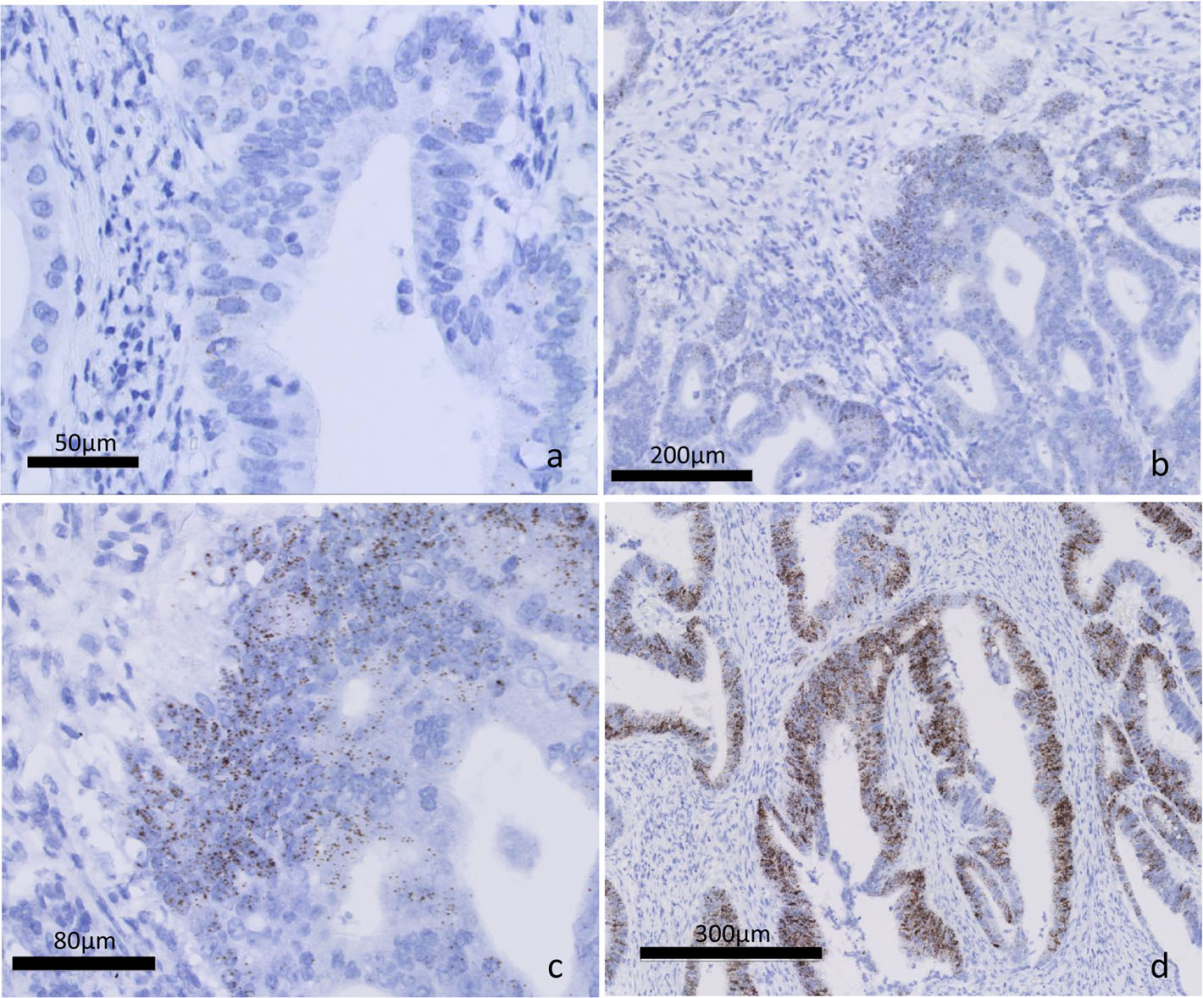
*LGR5* expression level was quantified according to the five-grade scoring system: score 2; a few dots per cell and no or very few dot; *LGR5* negative (**a**), score 3; > 10 dots per cell and < 10% positive cells overall; *LGR5* positive, (**b**, **c**), and score 4; > 10 dots per cell and > 10% positive cells; *LGR5* positive (**d**)

Formalin fixation -paraffin embedding and preservation of formalin-fixed paraffin-embedded (FFPE) tissues were performed according to College of American pathologists protocol (https://documents.cap.org/documents/practical-guide-specimen-handling.pdf). RNA-ISH was performed immediately after cutting sections from FFPE blocks. Positive staining was indicated by brown punctate dots in the nucleus and/or cytoplasm. The expression level of *LGR5* was quantified according to the five-grade scoring system previously described [8]: 0 = no staining or less than one dot per cell; 1 = 1 to 3 dots per cell; 2 = 4 to 10 dots per cell and no or very few dot clusters; 3 = > 10 dots per cell and < 10% positive cells overall; and 4 = > 10 dots per cell and > 10% positive cells with dot clusters. For a binary analysis, *LGR5* status was considered positive if the ISH score was > 2. (Fig. 1) [15] Immunohistochemical staining was performed using commercially available antibodies with the immuno-enzyme polymer method (Novolink Polymer Detection Systems for beta-catenin, Leica, Germany or Histofine Simple Stain MAX PO Multi for SATB2, Nichirei Biosciences, Tokyo, Japan) with 3, 3′-diaminobenzidone as the chromogen. The primary antibodies were used in accordance with the manufacturers’ instructions: beta-catenin (clone: EP35, EPITOMICS, Burlingame, California USA) and SATB2 (clone: EPNCIR130A, abcam, Cambridge, UK). Beta-catenin nuclear staining was classified as negative or scattered; no or very few scattered positive cells without any clusters, focal; positive cells clustered in focal areas, diffuse; positive cells distributed diffusely, homogeneously or heterogeneously in accordance with the previous study [16]. The extent of staining for SATB2 was scored semiquantitatively (no staining; < 5%; 5–25%; 26–50%; 51–75%; and 76–100%), and the maximum intensity was graded as negative, weak, moderate, or strong. For binary analyses, cases with 5% or more tumor cells showing moderate or strong intensity were considered positive [13].

Two of the authors (MI and JC) reviewed the RNA-ISH stains, and two of the authors (MI and HO) reviewed the immunohistochemical stains at a multiheaded microscope and reached a consensus score for each case.

### Statistics

Chi-square test or Fisher exact tests were used to characterize the relationship between categorical variables. Kruskal-Wallis chi-squared test and Mann–Whitney U-test were used for comparisons between *LGR5* expression level scores. Staining scores are non-parametric and are thus expressed as a median score with the interquartile range. Differences were considered to be significant at *p* < 0.05. All statistical analyses were performed with EZR (Saitama Medical Center, Jichi Medical University, Saitama, Japan), which is a graphical user interface for R (The R Foundation for Statistical Computing, Vienna, Austria).

## Results

The 29 patients with CAC included 18 with ulcerative colitis and 11 with Crohn’s disease. One patient with ulcerative colitis had synchronous carcinomas for a total of 30 tumors evaluated. A summary of the clinicopathologic features of the CAC cohort and sporadic controls is presented in Table 1. The median age at time of resection for patients with CAC was 54 years (range 26–76), significantly younger than sporadic cases (median age 67 years; range 40–95) (*p* = 0.017). CACs had a wider spectrum of tumor morphologies compared to sporadic CRCs. Only twelve of 30 (40%) CACs had conventional morphology compared to 80% of sporadic cases. None of sporadic cases were classified as LGTG. The one CAC case subclassified as “other” showed fetal enteric and hepatoid differentiation.

**Table 1:** Clinicopathologic features of study group patients

Clinical and pathologic features	CAC (N [%])	Sporadic CRCs (N [%])	***p***
No. of Cases	*30*	10	
Sex, male/female	19 / 11	5 / 5	0.482
Median age (range) (y)	54 (26–76)	67 (40–95)	0.017
Location			
colon	22 (73)	8 (80)	1
rectum	8 (27)	2 (20)	
pT stage			
1	8 (27)	1 (10)	0.144
2	*4 (13)*	2 (20)	
3	15 (50)	3 (30)	
4	3 (10)	4 (40)	
pN stage			
0	16 (53)	5 (50)	1
1 or 2	14 (47)	5 (50)	
Cancer subtypes			
Conventional	12 (40)	8 (80)	0.243
Mucinous	9 (30)	1 (10)	
Serrated	3 (10)	1 (10)	
LGTG	5 (17)	0 (0)	
Other	1 (3)	0 (0)	
*LGR5*			
Positive	9 (30)	9 (90)	0.002
(Score 3)	6 (20)	3 (30)	
(Score 4)	3 (10)	6 (60)	
Negative	21 (70)	1 (10)	
(Score 0)	2 (7)	0 (0)	
(Score 1)	9 (30)	0 (0)	
(Score 2)	10 (33)	1 (10)	

CAC indicates colitis associated colorectal adenocarcinoma

**Fig. 2:**
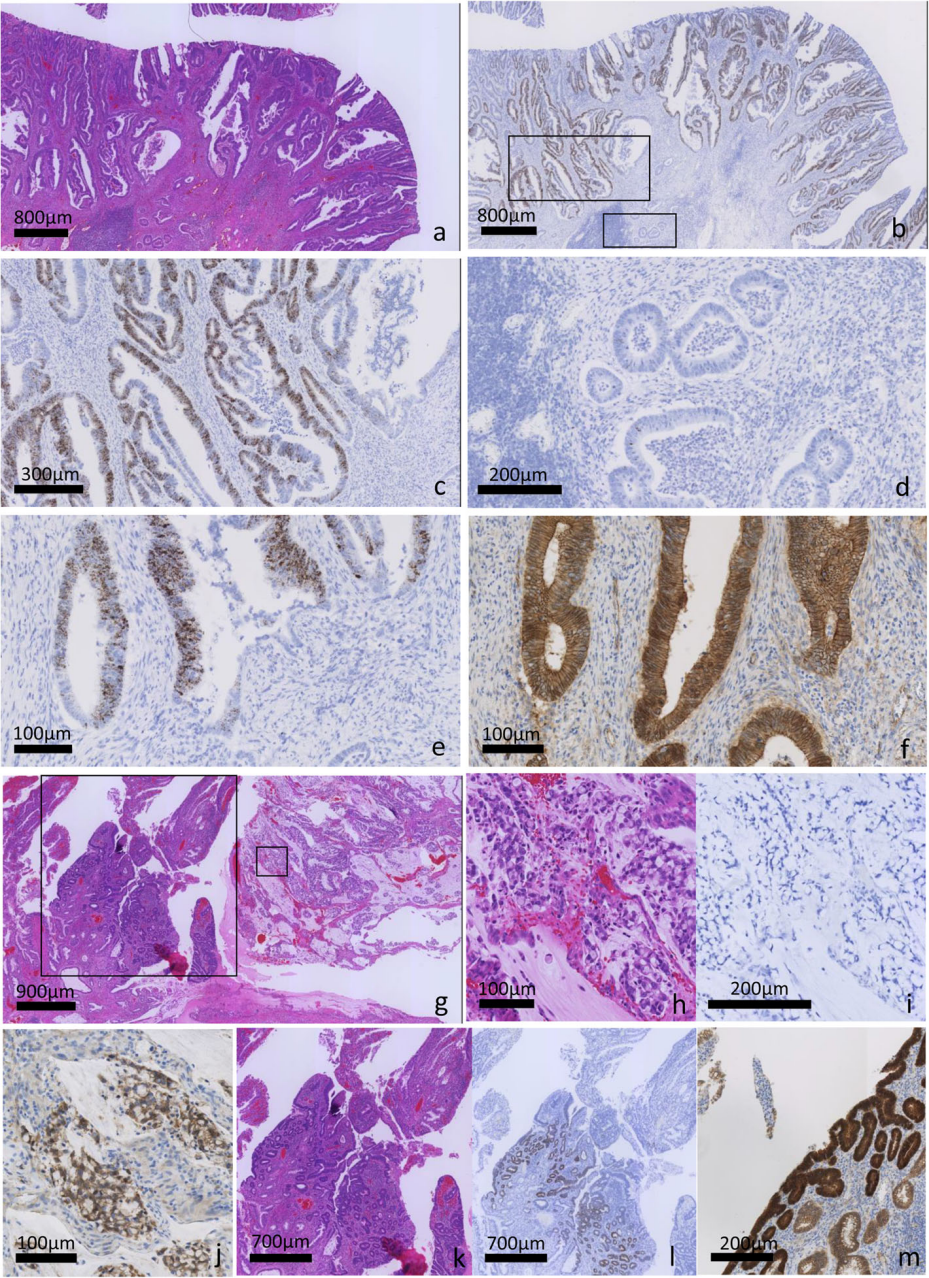
*LGR5* expression in colitis associated adenocarcinoma (CAC). CAC with conventional morphology (**a**) often showed diffuse and strong *LGR5* expression (**b** and **c**: high power view; score 4), however a few invasive glands adjacent to the *LGR5* positive area were *LGR5* negative. No morphological differences between these areas was apparent (**d**). An *LGR5* positive invasive component (**e**) showed nuclear translocation of beta-catenin (**f**). A high grade mucinous CAC had adjacent dysplasia with focal adenomatous morphology (**g**). The mucinous component was negative for *LGR5* (**h**); however, nuclear translocation of beta catenin was identified (**i**). Adjacent dysplasia with adenomatous morphology showed strong *LGR5* expression (**l**) and nuclear expression of beta catenin (**m**)

**Fig. 3:**
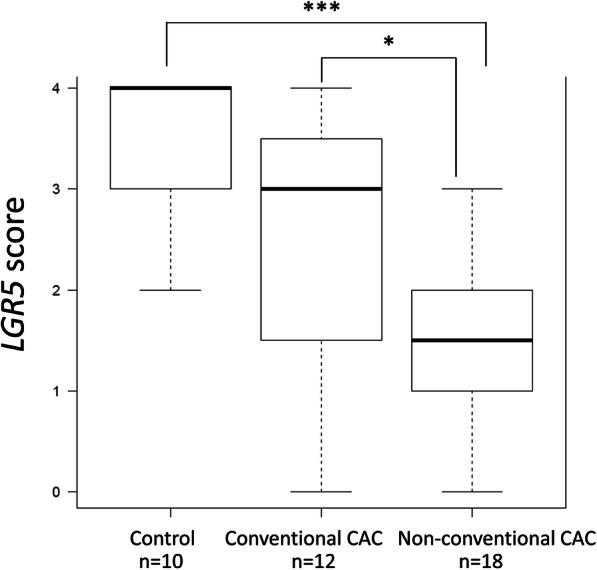
Expression level of *LGR5* in control sporadic colorectal adenocarcinoma, conventional type colitis associated adenocarcinoma and non-conventional type colitis associated adenocarcinoma. *P = 0.034, ***P < 0.001

Adjacent non-neoplastic colonic mucosa was examined as an internal control for *LGR5* expression. In all cases, there were measurable brown dots in the base of crypts in control areas. *LGR5* was positive in 30% (9/30) of CAC cases (median 2.0, interquartile range 1.00–3.00) and 90% (9/10) of sporadic CRCs (median 4.0, interquartile range 3.00–4.00) (*p* = 0.002). In CACs, the distribution of *LGR5* expression was heterogenous in the overlying mucosa and in some cases, overlying or adjacent dysplasia showed higher expression than the invasive component. An abrupt transition to *LGR5* expression was identified in the CAC or dysplasia component in some cases. (Fig. 2) A large majority (89%) of *LGR5* positive CAC cases were of the conventional histologic type, while morphologies were much more variable in the *LGR5* negative CAC cases. (Fig. 2) In conventional type CACs, eight of 12 cases (67%) were *LGR5* positive (median 3.0; interquartile range 1.75–3.25) and in contrast, only one of 18 (6%) non-conventional type CACs (one with serrated morphology) was *LGR5* positive (median 1.5; interquartile range 1.00–2.00) (*p* < 0.001). Conventional type CAC showed a significantly higher level of *LGR5* expression than non-conventional type CAC (*p* = 0.034). Conventional type CACs showed a lower level of *LGR5* expression than sporadic CRCs, but this did not reach statistical significance (*p* = 0.221), whereas, expression level of *LGR5* in non-conventional type CACs was significantly lower compared to sporadic CRCs (*p* < 0.001). (Fig. 3).

In CACs, *LGR5* expression was associated with nuclear beta catenin expression, but not with SATB2 expression; only two of 21 (10%) *LGR5* negative CACs showed diffuse nuclear beta-catenin expression, while 6 of 9 (67%) *LGR5* positive CACs showed diffuse nuclear-beta catenin expression (*p* = 0.003). Eleven of 30 CACs (37%) retained SATB2 expression and the expression rate was 33 and 44% in *LGR5* positive CACs and *LGR5* negative CACs, respectively (*p* = 0.687).

*LGR5* positive CACs were more likely to be located in the rectum (*p* = 0.032). There was no significant difference in pT stage, pN stage, IBD phenotype, age, or sex between *LGR5* positive CACs and negative CACs. (Table 2).

**Table 2:** Clinicopathologic Features of *LGR5* positive CACs and *LGR5* negative CACs

	*LGR5 *positive CACs (N [%])	*LGR5* negative CACs (N [%])	p
No. of Cases	*9*	21	
Sex, male/female	5 / 4	14 / 7	0.687
Median age (range) (y)	53 (24–76)	54 (31–65)	0.557
Location			
colon	4 (44)	18 (86)	0.032
rectum	5 (56)	3 (14)	
pT stage			
1	4 (44)	4 (19)	0.471
2	*1 (11)*	3 (14)	
3	4 (44)	11 (52)	
4	0 (0)	3 (14)	
pN stage			
0	6 (67)	10 (47)	0.440
1 or 2	3 (33)	11 (52)	
Cancer subtypes			
Conventional	8 (89)	4 (19)	< 0.001
Mucinous	0 (0)	9 (42)	0.029
Serrated	1 (11)	2 (10)	1
LGTG	0 (0)	5 (24)	0.286
Other	0 (0)	1 (5)	1
Immunohistochemistry			
Beta- catenin nuclear expression			
Diffuse	6 (67)	2 (10)	0.003
Negative/ Scattered or Focal	3 (33)	19 (90)	
SATB2	4 (44)	7 (33)	0.687

CAC indicates colitis associated colorectal adenocarcinoma

## Discussion

Here, we evaluated the *LGR5* expression profile of 30 CAC cases and 10 sporadic CRC cases and identified that *LGR5* was less frequently expressed in CACs than in sporadic CRCs. Further, we found CAC with non-conventional morphology showed significantly lower *LGR5* expression levels and nuclear translocation of beta-catenin than the conventional subtype of CAC.

LGR5 has been considered one of the most reliable crypt stem cell markers of CRCs, particularly those that arise through the *APC*-mutant pathway of tumorigenesis [9]. LGR5 is reportedly overexpressed in human colorectal adenomas and cancers by immunohistochemistry [6, 17, 18] and ISH [15], and Martin et al. showed that the mRNA levels of *LGR5* expressed in human CRCs is 10-fold expanded than normal intestinal crypt [19]. Jang et al. recently showed that *LGR5* mRNA positivity is observed in 68% of 788 human CRCs and suggested positive correlation with the chromosomal-instability pathway (characterized by left-sided location and nuclear β-catenin expression; representing an abnormal *Wnt* signal activation) and negative correlation with MSI, CIMP-high, and BRAF mutations [15]. In accordance with these findings, we found positive *LGR5* expression in 90% of our sporadic CRC control group. However, only 30% of CAC were *LGR5* positive. The latter finding is in agreement with the previous study by Yasuda et al. reporting significantly less expression of reliable intestinal stem cell markers such as CD133 [20], OCT4 [21] and NANOG [22] in CAC than sporadic CRC [23]. The molecular pathogenesis of CAC is different than that of sporadic CRCs with genomic changes that appear directly linked to the effects of ongoing inflammation and repeated mucosal injury in the IBD [11, 12]. In keeping with this, we recently reported CAC tends to show non-conventional tumor morphology and loose intestinal markers such as SATB2 and CDX2, and show aberrant gastric mucin expression [13], Ishibashi et al. described the expression profile of Atonal homolog-1 (ATOH1), a master transcription factor of the secretory lineage of intestinal epithelial cells, which retain their potential to revert to intestinal stem cells [24–26] in colitic mucosa. They identified both ATOH1 and LGR5 double positive tumor cells as well as LGR5 single positive tumor cells, suggesting that colitic tumors are mosaic and consisted of a heterogenous population of tumor stem cell [27]. Our data also support this interpretation given the heterogeneous distribution of *LGR5* expression in the dysplastic and invasive components of some cases. While the exact mechanism of decreased *LGR5* expression in CAC is uncertain, these data demonstrate that CACs have a unique expression profile of intestinal stem cell markers.

We found that despite only 40% of CACs in our cohort showing a conventional morphology, 89% of *LGR5* positive CAC were conventional in appearance. These also tended to be located in the rectum and associated with nuclear beta-catenin staining. This raises the possibility that the minority of CACs in our cohort that showed conventional morphology might have arisen through the conventional *APC* pathway. However, as the majority of tumors had non-conventional morphology and were also *LGR5* negative, our data provide support to other recent studies demonstrating that most CAC cases do not arise through the conventional *APC* pathway [12, 28]. Although it did not reach statistical significance, conventional type CACs trended toward a lower level of *LGR5* expression compared to the control group of sporadic CRCs. Thus, even in tumors arising through more conventional molecular pathways in the setting of colitis, *LGR5* expression might be affected by ongoing inflammation and repeated mucosal injury. This hypothesis is supported by other studies showing that Lgr5+ stem cells are highly sensitive to epithelial injury induced by radiation or colitis [27, 29, 30].

Our results in human cancers do not entirely align with previous data from animal models of colitis. Kim et al. reported increased *Lgr5* expression in an AOM / DSS - colitis mouse model. They showed that *Lgr5* expression is gradually increased as tumors developed with repeated colitis and reported all dysplastic lesions and cancers showed high *Lgr5* expression [10]. This discrepancy could be due to differences between human CAC and the murine model. Interestingly, the figure showing tumor morphology in their report shows a more conventional morphology, implying that the murine model may simulate the carcinogenesis pathway in the conventional type of human CAC.

Data regarding any potential prognostic significance of LGR5 expression in sporadic CRC are mixed. Recent studies using RNA-ISH for *LGR5* evaluation reported that *LGR5* expression is an independent predictor of favorable outcome in CRCs [15, 31]. On the contrary, a previous meta-analyses of immunohistochemical studies of LGR5 expression showed that high LGR5 expression is associated with shorter overall survival and disease free survival [32, 33].

Kazama et al. described the immunohistochemical expression profile of LGR5 in ulcerative colitis cases and showed increased LGR5 expression in dysplasia and CAC [34]. This result is contrary to our present study, but may be due to the different methods of LGR5 detection, as the reliability of antibodies against LGR5 remains uncertain [35, 36], and most recent studies have used ISH to detect *LGR*5 expression.

One limitation of our cohort is the limited number of cases and the unavailability of clinical follow up data as well as further molecular analysis. Additional studies using a larger cohort will be needed to determine the relationship between LGR5 expression and clinical prognosis in human CACs.

## Conclusion

Unlike sporadic CRC, LGR5 expression, along with nuclear beta-catenin expression is infrequent in CAC, particularly those with non-conventional morphology. It is more commonly expressed in CAC with conventional morphology and in tumors located in the rectum, indicating that a limited subset of CACs may arise through the conventional *APC* pathway. While the precise mechanisms for the low frequency of LGR5 expression in CAC remains undefined, the wider spectrum of tumor morphology in CAC may be associated with absence of a *LGR5*-expressing intestinal stem cell phenotype.

## Data Availability

All data generated and analyzed during the current study are available from the corresponding author on reasonable request.

## Abbreviations

LGR5: Leucine-rich repeat-containing G-protein-coupled receptor 5
CRC: Colorectal adenocarcinoma
CAC: Colitis associated colorectal adenocarcinoma
LGTG: Low grade tubuloglandular
